# Precise Tuning of Flexoelectricity in SrTiO_3_ by Ion Irradiation

**DOI:** 10.1002/advs.202411391

**Published:** 2024-12-16

**Authors:** Shuai Nan, Shipu Xu, Yunlong Ren, Xin Yuan, Yuhua Ma, Pengfei Zhai, Fuxiang Zhang

**Affiliations:** ^1^ Songshan Lake Materials Laboratory Dongguan Guangdong 523808 China; ^2^ Institute of Physics Chinese Academy of Sciences Beijing 100190 China; ^3^ School of Microelectronics Science and Technology Sun Yat‐sen University Zhuhai Guangdong 519082 China; ^4^ Guangdong Provincial Key Laboratory of Quantum Engineering and Quantum Materials School of Physics South China Normal University Guangzhou Guangdong 510006 China; ^5^ Guangdong‐Hong Kong Joint Laboratory of Quantum Matter Frontier Research Institute for Physics South China Normal University Guangzhou Guangdong 510006 China; ^6^ Institute of Modern Physics Chinese Academy of Sciences Lanzhou Gansu 730000 China

**Keywords:** flexoelectricity, ion radiation, quantized collision model, strain gradient modulation

## Abstract

Flexoelectric coefficient is a tetradic and its introduction enables centrosymmetric materials to exhibit piezoelectricity. However, the flexoelectric paradigm currently lacks a strategy to effectively tune the strain gradient for optimal electro‐mechanical coupling. This study proposes a quantized collision model accessible through ionic irradiation technology to explore the flexoelectricity and precisely modulate the strain gradient. The lattice strain is introduced in SrTiO_3_ (STO) single crystals and tuned broadly by irradiation with ions of He^+^, C^+^, and P^+^ at dose of 1 × 10^14^ and 2 × 10^15^ ion cm^−2^, respectively. Under C^+^ ion irradiation at a dose of 2 × 10^15^ ion cm^−2^, thin‐film X‐ray diffraction reveals a strain gradient up to ≈0.65% nm^−1^. The resulted polarization is found to orient out‐of‐plane, as observed through X‐ray reciprocal space mapping and high‐angle annular dark field scanning transmission electron microscopy. Piezoresponse force microscopy characterization reveals that the electric‐induced out‐of‐plane polarization reversal emerges at room temperature, corresponding to a stain gradient ≈0.05% nm^−1^ in STO's flexoelectric response. This study demonstrates that ion irradiation is an effective strategy for precisely tuning the flexoelectric properties.

## Graphical abstract

In this work, polarization and flexoelectricity of SrTiO_3_ are induced by ion irradiation at room temperature. Ion irradiation generates strain and polarization along the out‐of‐plane direction, with both the magnitude of the strain gradient and the depth being controllable. Local lattice strain is also observed at the atomic‐level scale using high‐resolution TEM.

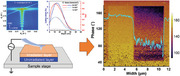

## 1 Introduction

Piezoelectricity in crystalline materials lacking inversion symmetry generates an electric response to applied mechanical stress, leading to the development of advanced piezoelectric devices that extend beyond Moore's law.^[^ ^1^, ^2^, ^3^ ^]^ The electro‐mechanical coupling mechanism is manifested as either ferroelectricity or flexoelectricity.^[^ ^4^, ^5^ ^]^ Polarization is introduced in both paradigms as an order parameter in the electric‐induced phase transitions.^[^ ^6^, ^7^ ^]^ Featuring with spontaneous polarization, ferroelectric materials with non‐centrosymmetry are widely integrated into piezoelectric devices.^[^ ^8^, ^9^, ^10^ ^]^ However, due to the limited ferroelectric materials available, the flexoelectric paradigm is proposed to expand the material library.^[^ ^5^, ^11^, ^12^ ^]^ This is because that the flexoelectric coefficient is a tetradic parameter, and allows for the inclusion of centrosymmetric materials with strain gradients.^[^ ^5^, ^13^ ^]^

**Figure 1:**
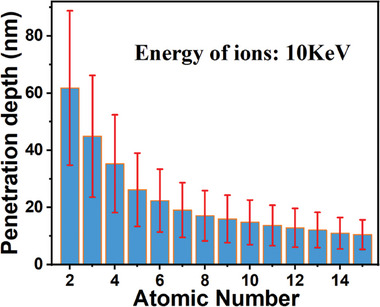
The calculated penetration depth in an STO crystal irradiated with 10 keV ions with atomic number ranging from 2 to 15 (He^+^‐P^+^).

In the flexoelectric paradigm, the flexoelectric coefficient is related to the strain gradient in the bulk which can be introduced in diverse ways.^[^ ^5^ ^]^ Conventional methods include interface engineering,^[^ ^14^ ^]^ external mechanical stress,^[^ ^13^, ^15^ ^]^ nano engineering,^[^ ^16^, ^17^ ^]^ element doping,^[^ ^18^ ^]^ and compositional gradient introduction.^[^ ^19^ ^]^ These methods enable the introduction of strain gradients to the lattice and stoichiometric modulation across the bulk.^[^ ^5^ ^]^ The lattice mismatch between the substrate and epitaxial film can generate large lattice strains in the interface and introduce polarization within the film. However, the strain at the interface is usually confined to a very thin layer, often a few atomic layers,^[^ ^20^ ^]^ and is also limited by the selection of substrates and film materials. Polarization of centrosymmetric materials, induced by external stress,^[^ ^15^ ^]^ is a reversible process within the elastic deformation regime, meaning the polarization disappears once the external force is released. In nanomaterials, polarization is primarily due to surface effect^[^ ^16^ ^]^ and this phenomenon is difficult to achieve in bulk materials.^[^ ^17^ ^]^ Flexoelectricity, which arises from element doping and compositional gradient, requires precise control of composition at the atomic scale, as well as complicated deposition process and specialized facilities.^[^ ^19^ ^]^ To distinctly enhance the piezoelectric effect, a strategy assisted by helium implantation is reported to independently alter the out‐of‐plane lattice constant.^[^ ^21^, ^22^ ^]^ To further refine the flexoelectricity, it is essential to propose strategies capable of precisely tuning strain gradients only along the out‐of‐plane direction. Compared to conventional methods,^[^ ^2^ ^]^ ion irradiation offers great control over the domain and magnitude of strain gradient in centrosymmetric materials by adjusting the mass, energy, and dose of the incident ions. The extent of damage caused by ion irradiation in the target materials depends on the energy transfer between the ions and nuclei, as well as the electrons in the material. The penetration depth is determined by the mass and energy of incident ions, as well as the composition of the target material. Generally, lighter ions can penetrate to greater depth when the ion energy is fixed. The penetration depth can be calculated using the Stopping and Range of Ions in Matter (SRIM) software package.^[^ ^23^ ^]^ **Figure** **1** illustrates the calculated penetration depth in an STO crystal irradiated with 10 KeV ions, ranging from He^+^ to P^+^ (*Z* = 2–15). However, it is important to ensure that the introduction of strain gradients does not result in severe lattice damage, such as the destruction or amorphization of the lattice, which cannot be predicted directly from theoretical calculation.

This study applies ion irradiation technology to explore flexoelectricity and aims to precisely control the strain gradient along the out‐of‐plane direction in STO. By varying both the mass of ions and irradiation dose, the lattice strain in STO is adjusted from ≈0.05% to 0.65% nm^−1^. High‐angle annular dark field scanning transmission electron microscopy (HAADF‐STEM) reveals that the resulted polarization orients out‐of‐plane, consistent with the X‐ray reciprocal space mapping measurements. Electric‐induced polarization reversal emerges as the strain gradient exceeds 0.05% nm^−1^ at room temperature by piezoresponse force microscopy (PFM) characterization. Our study demonstrates that ionic irradiation is an effective strategy to tune the strain gradient and flexoelectricity in materials.

## 2 Results

### 2.1 Ionic Irradiation Quantized Collision Model

**Figure 2:**
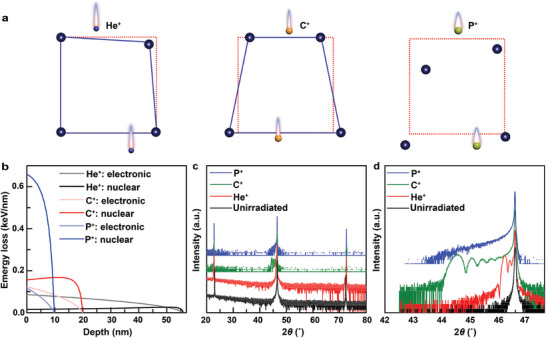
Structural evolution for the ion‐irradiated SrTiO_3_. The STO crystals are irradiated by 10 keV ions with a dose of 1 × 10^15^ ions cm^−^2. a) The schematic diagrams of atomic displacements induced by He^+^, C^+^, and P^+^ incidence. b) The calculated energy losses and penetration depths in STO for different ions. Compared with He^+^ ions and P^+^ ions, C^+^ ion has the moderate energy loss and penetration depth. c) The XRD patterns measured with a symmetric scan. d) High‐resolution *θ‐2θ* scans of the virgin and irradiated STO around the (200) lattice plane.

Flexoelectricity is characterized with strain gradient across the bulk.^[^ ^5^, ^24^ ^]^ This work applies ion irradiation to induce strains in SrTiO_3_. **Figure** **2**a schematically illustrates three identical cases of lattice response by incident ions of He^+^, C^+^, and P^+^, aiming at precisely constructing lattice strains in STO. By tailoring irradiation, such as C^+^ incidence, the strain is characterized by an out‐of‐plane gradient, rather than amorphization in the case of P^+^ bombardment presented in Figure 2a. To demonstrate the strain induced by ion irradiation, we employ a quantized collision model. The lattice ions of STO create periodic coulomb potentials, featuring with a potential barrier represented by delta (αδ(*x*)). This coulomb potential divides the collision into two zones, i.e., the zone placed before collision (Zone I) and other situated after the collision (Zone II). In Zones I and II, the time‐independent Schrödinger equation for the collision system is presented below:

(1) $$E\Psi \left(x\right)=-\frac{\hbar^{2}}{2\mu }\frac{d^{2}}{dx^{2}}\Psi \left(x\right)$$

where the *E*, Ψ(x), ℏ, *µ*, and x are respectively the energy of the incident ion, the wave function of the collision system, reduced Planck constant, reduced mass, and the distance between the incident ion and the STO barrier. The probability of transmission found in Zone II is estimated by the following equation:

(2) $$P=\frac{1}{1+\beta^{2}}$$

where β is correlated with the mass of the irradiated ion by the Equation (3):

(3) $$\beta =\alpha \;{\left(2E\right)}^{-\frac{1}{2}}{\left(\mu \right)}^{\frac{1}{2}}$$

where α is a constant. *P* is negatively correlated with μ, and this method illustrates that the incident depth is negatively related to the mass of ions.

To demonstrate the quantized collision model, the simulated calculation about energy loss is performed by the SRIM package^[^ ^23^ ^]^ and the atomic displacement is extracted by the simulated nuclear energy loss for different ionic irradiation conditions. According to the threshold of thermal spike model,^[^ ^25^, ^26^ ^]^ a total energy loss larger than 10 keV/nm is required for significant damage formation, e.g., the creation of an ion track.^[^ ^27^, ^28^ ^]^ In the cases of He^+^, C^+^, and P^+^ with 10 keV energy, the calculated electronic energy losses in STO are at most 0.12 keV nm^−1^, which is well below the threshold (Figure 2b), indicating a trivial effect for the atomic displacement. Meantime, the electronic energy losses are also dramatically lower than the value (>0.43 keV nm^−1^) needed to anneal the pre‐irradiated damage in STO.^[^ ^29^ ^]^ However, the nuclear energy losses are up to 0.03 , 0.17 , and 0.65 keV nm^−1^ for He^+^, C^+^_,_ and P^+^ ions, respectively. In comparing the integral of nuclear energy loss, the value obtained from P^+^ irradiation is up to 5.04 keV concentrated within ≈10 nm, which is greater than the 3.04 keV for C^+^ irradiation dispersed over ≈20 nm, and 1.15 keV for He^+^ irradiation deposited within ≈60 nm. As a result, irradiation with P^+^ ions causes the most severe lattice damage, while He^+^ ions cause the least damage to the lattice of STO. Figure 2a schematically illustrates the different levels of lattice damage induced by these ions. Obeying the quantized collision model, the incident depth is demonstrated to be negatively correlated with the mass of ions, where the He^+^ is of the deepest depth of ≈60 nm. Being of the mass‐dependent radiation depth, the different nuclear energy losses result in distinct variations in the atomic displacements in STO caused by P^+^, C^+^, and He^+^ ion irradiation.

In order to reveal the damage, both ion‐irradiated and virgin STO were measured with X‐ray diffraction measurements in θ‐2θ scan mode. The observation of only (*h*00) lattice planes suggests a high quality of the single crystal (Figure 2c). Lattice strains induced by atomic displacement from ion irradiation are measured using high‐resolution θ‐2θ scans near the (200) Bragg peak. Besides the sharp (200) diffraction from the unirradiated region, fringe peaks are detected in the He^+^ and C^+^ irradiated samples (Figure 2d). These fringe peaks are attributed to the interference of the non‐symmetric distribution of lattice strains around the peak damage. The fringe peaks start ≈46.1° in the He^+^ irradiated STO at a dose of 1 × 10^15^ ions cm^−2^, and the lattice strain with a rough estimation is ≈1%.^[^ ^30^ ^]^ For the C^+^ irradiated STO at the same dose, the fringe peak begins ≈44.5°, which indicates that the lattice stain is enhanced by C^+^ irradiation. In contrast, P^+^ irradiation at a dose of 1 × 10^15^ ions cm^−2^ resulted in the elimination of observable strains due to severe lattice damage. The diffused shoulder around the (200) plane with negligible fringe peaks indicates the existence of aperiodic phases (Figure 2d). The aperiodic structure with no observable lattice strain has also been reported in irradiated ceramics of zircon^[^ ^31^ ^]^ and apatite.^[^ ^32^ ^]^

### 2.2 Lattice Strain Tuned by C^+^ Doses

**Figure 3:**
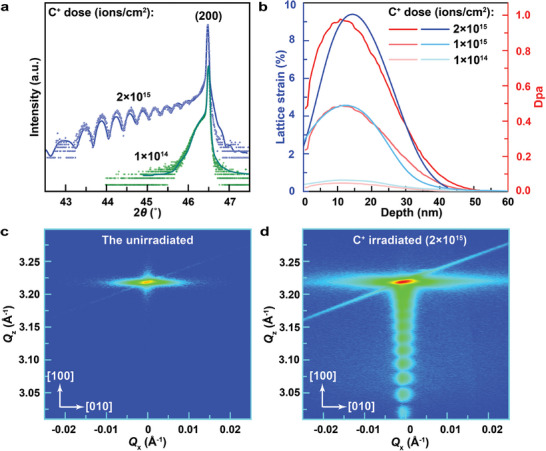
Strain profile and orientation induced by 10 keV C^+^ irradiation at different doses. a) The starting of fringe peaks shifts with the radiation dose. The initial peak decreases from ≈46.3° to ≈44.8° as the radiation dose increases from 1 × 10^14^ to 2 × 10^15^ ions cm^−2^. b) The simulated lattice strain profiles at different incidence doses. Extracted from the experimental XRD patterns, the maximum lattice strain increases from ≈0.6% to ≈9.4% as the dose increases to 2 × 10^15^ ions cm^−2^, and the gradient distributions of lattice strains closely match with DPAs. c,d) The reciprocal space mapping for unirradiated (c) and irradiated STO (d). There are no fringe peaks in both in‐plane and out‐of‐plane directions of the unirradiated STO, C^+^ ions irradiation at a dose of 2 × 10^15^ ions cm^−2^ induced fringe diffractions along the *Q_z_* but not *Q_x_* axis which suggests the existence of out‐of‐plane lattice strain only.

In order to study the dependency of lattice strain on ion dose, C^+^ ion is used with doses ranging from 1 × 10^14^ to 2 × 10^15^ ions cm^−2^. As shown in **Figure** **3**a, symmetric X‐ray scans find that the fringe peaks start at 2*θ* position of ≈46.3° for the irradiation dose of 1 × 10^14^ ions cm^−2^ and shift dramatically to ≈42.8° for a dose of 2 × 10^15^ ions cm^−2^. This shift in 2*θ* suggests a significant enhancement of lattice strain in an incident dose of 2 × 10^15^ ions cm^−2^, which is roughly estimated to be ≈8.2% relative to the unirradiated STO.^[^ ^30^ ^]^

The lattice strain depth profiles of the irradiated STO are quantitively derived from the simulation of the measured XRD patterns using the software RaDMaX.^[^ ^33^ ^]^ As shown in Figure 3b, the lattice strain curve enhances as the incident dose increases from 1 × 10^14^ to 2 × 10^15^ ions cm^−2^. The dose of 2 × 10^15^ ions cm^−2^ produces the maximum lattice strain of ≈9.4% at a depth of ≈14 nm, which is compatible with the estimated value (≈8.2%) by the lowest 2*θ* angle of the fringe peaks (Figure 3a). The maximum strain decreases from ≈4.6% down to ≈0.6% when the incident dose is reduced from 1 × 10^15^ to 1 × 10^14^ ions cm^−2^. The gradient depth distribution of radiation‐induced lattice strains closely matches the calculated damage distribution. The distinct characteristic of damage distribution from energetic ions is the existence of the diffused scattering near the Bragg peaks.^[^ ^34^, ^35^ ^]^ The calculated damage level of C^+^ bombardment in STO is up to 0.98 displacement per atom (DPA) for the dose of 2 × 10^15^ ions cm^−2^, which is higher than those for lower doses.

The C^+^‐incident‐induced lattice strain is featured with strength and orientation. X‐ray reciprocal spacing mappings (RSM) of both non‐irradiated STO and irradiated with C^+^ at a dose of 2 × 10^15^ ions cm^−2^ are shown in Figure 3c,d, respectively. Similar to the 1D measurements in Figure 2d, the C^+^‐ion‐irradiated sample triggers fringe diffractions along the *Q*_z_ direction (Figure 3d), which is directed out‐of‐plane. In contrast to the non‐irradiated sample, no diffuse scattering is observed along the *Q*_x_ axis, indicating that there are no distinct in‐plane strains and that radiation‐induced lattice strain is along the out‐of‐plane direction only, and similar results were observed in other materials before.^[^ ^30^ ^]^

### 2.3 TEM Observation of Ion‐Irradiation‐Induced Lattice Strains

**Figure 4:**
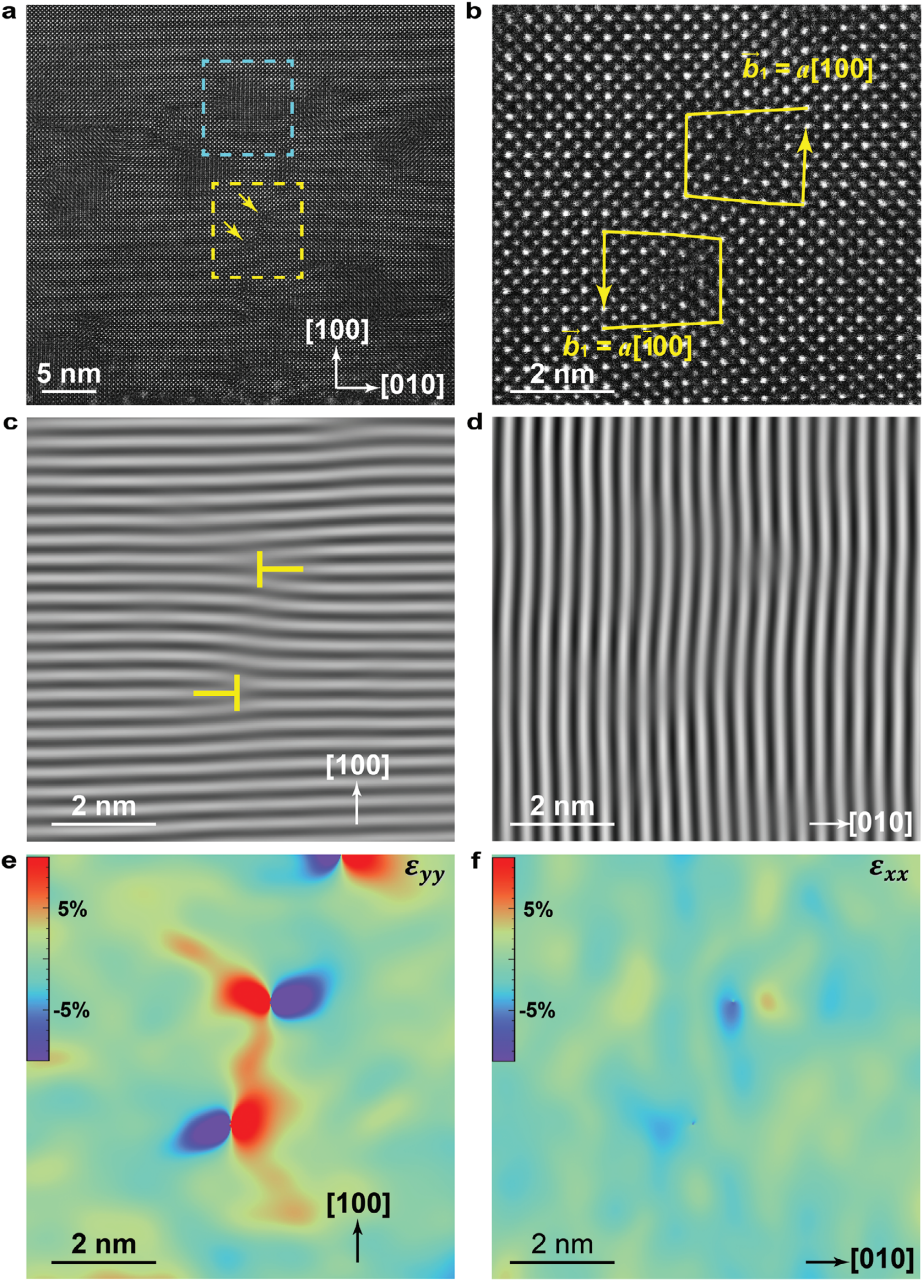
Atomic‐scale observation of lattice strains induced by C^+^ ions. Irradiated by 10 keV C^+^ ions with 2 × 10^15^ ions cm^−2^, HRTEM image reveals that the strain concentrates mainly in the out‐of‐plane direction. a) HAADF‐STEM image of the irradiated zone. The bottom is the irradiated surface, which shows that C^+^ ions irradiation induces many edge dislocations (represented by two yellow arrows). b) The magnified image from the yellow square in a). This image illustrates two adjacent edge dislocations with the Burgers vectors of ${\overset{⇀}{b}}_{1}$= a[100] and ${\overset{⇀}{b}}_{2}$= a[$\bar{1}$00], i.e., both paralleling to the irradiation direction. The Bragg filtered image of c) (100) plane and d) (010) plane shows that all radiation‐induced edge dislocations lie in the out‐of‐plane direction, marked by yellow “┬”, but not the in‐plane direction. e) The radiation‐induced lattice strains (including tension and compression) concentrated around the dislocations in the out‐of‐plane direction. f) The minimal lattice strains appearing toward the in‐plane direction around the distorted area.

Atomic‐scale observation of irradiation‐induced defects and lattice strains is conducted using cross‐sectional TEM from the [001] zone axis. To maximize the probability of detecting the origins of lattice strains induced by ion irradiations from atomic‐scale, this work focuses on STO irradiated with 10 keV C^+^ at a high dose of 2 × 10^15^ ions cm^−2^, which contains more pronounced lattice strains compared to other radiation scenarios (see Figure 3b). In the HAADF‐STEM image (**Figure** **4**a), the blue and yellow squares highlight the heavily strained zone and edge dislocations induced by C^+^ ion irradiation, respectively. Figure 4b zooms in on two typical adjacent edge dislocations (magnified from the yellow square in Figure 4a) oriented in opposite orientations. One edge dislocation has a Burgers vector (${\overset{⇀}{b}}_{1}$) of *a*[100], and the other has a Burgers vector (${\overset{⇀}{b}}_{2}$) of *a*[$\bar{1}$00], both parallel to the irradiation direction. Here, *a* is the lattice constant of the conventional unit cell of STO.

In the Bragg‐filtered analysis of the HAADF‐STEM image, all the edge dislocations manifest out‐of‐plane, aligning parallel to the path of ion penetration rather than in‐plane. In Figure 4c, representative edge dislocations are marked by yellow “┬” symbols in the Bragg‐filtered image of the (100) plane corresponding to the yellow areas in Figure 4b. Note that the dislocation pairs with Burgers vectors *a*[100] and *a*[$\bar{1}$00] are likely to form simultaneously. On the contrary, radiation‐induced dislocations revealed by the Bragg‐filtered image of the (010) plane are minimal in the in‐plane direction (Figure 4d), and lattice distortions can be largely ignored. The concentration of edge dislocations in out‐of‐plane lattice is due to the tensile strain induced by the incident ions.

Using geometric phase analysis,^[^ ^36^ ^]^ Figure 4e illustrates the distribution of strain fields in the out‐of‐plane direction, i.e., perpendicular to the (100) plane. For a strained lattice in epitaxial films, polarization can be visualized by phase‐field simulations based on the HAADF‐STEM image.^[^ ^37^ ^]^ However, for ion‐irradiated STO crystals, deriving the atomic displacement vector map from the observed HAADF‐STEM images at the atomic scale is challenging due to lattice imperfections caused by irradiation‐induced defects. Each red‐blue halo in the strain field image (Figure 4e) corresponds to a strain field zone calculated from the core of the edge dislocations shown in Figure 4b,c. It indicates that irradiated‐induced strain predominantly concentrates in these regions.

Figure 4f shows the minimal in‐plane lattice strains at the distorted area (Figure 4b), which is likely caused by the ≈7° offset of [100] zone axis from the ion irradiation direction. Ion irradiation primarily induces out‐of‐plane lattice strains, which has also been previously reported in yttria‐stabilized ZnO,^[^ ^30^ ^]^ La_0.7_Sr_0.3_MnO_3_ thin film,^[^ ^22^ ^]^ LiTaO_3_,^[^ ^38^ ^]^ and STO.^[^ ^39^ ^]^

### 2.4 Radiation‐Induced Flexoelectricity

**Figure 5:**
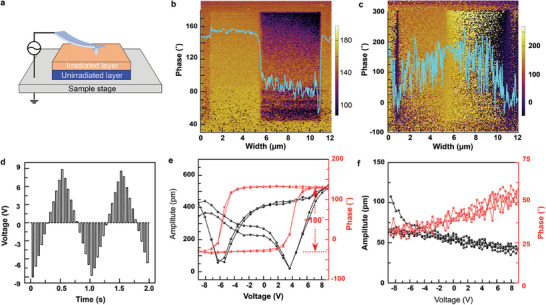
Polarization switching in irradiated STO. a) The schematic diagram for PFM characterization. b) PFM phase contrast image in the out‐of‐plane direction and c) in‐plane direction. d) The voltage pulses for inducing the polarization switching. e) The electric hysteresis and amplitude spectroscopy loops in the out‐of‐plane direction and (f) in‐plane direction.

Ion irradiation with C^+^ tailors the lattice strain in STO with respect to both magnitude and orientation to optimize flexoelectricities. To demonstrate the flexoelectric effect, STO irradiated with 10 keV C^+^ ions at a dose of 2 × 10^15^ ions cm^−2^ undergoes vector PFM measurements (**Figure** **5**a). PFM characterize polarization switching hysteresis loop and piezoelectric domains in ion irradiated STO, comparing with the unirradiated sample.

Figure 5b,c depict the phase‐contrast PFM images of the out‐of‐plane and the in‐plane phases, respectively, and the irradiated STO is pre‐scanned with an external voltage ranging from +8 to −8 V. In the out‐of‐plane phase characterization, a distinct phase contrast is presented, with an estimation of variation up to ≈80° observed in the profile view (Figure 5b). On the contrary, the in‐plane phase mapping shows trivial phase contrast (Figure 5c), indicating that the flexoelectric effect in STO predominantly involves out‐of‐plane polarization. For unirradiated STO, phase contrast is minimal in both out‐of‐plane and in‐plane phase mappings (Figure S2a,b, Supporting Information). Furthermore, the flexoelectric effect induced by C^+^ irradiation in STO is investigated through piezoelectric hysteresis loops shown in Figure 5d–f. Initially, a series of bias pulses pre‐polarized toward the STO sample, varying from −9 to +9 V, and the phase hysteresis loop is extracted for both out‐of‐plane and in‐plane polarizations (Figure 5d). The out‐of‐plane phase hysteresis exhibits ≈180° polarization reversal with a characteristic butterfly‐shaped amplitude loop (Figure 5e), while the in‐plane polarization shows no obvious hysteresis loop (Figure 5f). In contrast, the hysteresis loops of the unirradiated STO show no polarization reversals or butterfly shapes in both out‐of‐plane (Figure S2c, Supporting Information) and in‐plane directions (Figure S2d, Supporting Information). This finding confirms that the C^+^ irradiation induces flexoelectric behavior in STO, with polarization orientated out‐of‐plane.

As reducing the dose of C^+^ ions to 1 × 10^14^ ions cm^−2^, the irradiated STO still shows distinct phase contrast in the out‐of‐plane direction as illustrated in Figure S3a,b (Supporting Information), where the phase contrast is estimated to be ≈150°. This contrast is attributed to a strain gradient of ≈0.05% nm^−1^ originated from the C^+^ irradiation. Correspondingly, the polarization hysteresis loops further confirm the flexoelectric effect in STO irradiated with this low dose of 1 × 10^14^ ions cm^−2^. Figure S3c (Supporting Information) shows the polarization reversal of ≈180° and the amplitude loop with a butterfly shape in the out‐of‐plane direction. However, in the in‐plane direction, there is no stable polarization reversal and butterfly‐shaped amplitude loop observed (Figure S3d, Supporting Information).

## 3 Conclusion

This investigation has successfully demonstrated the induction of flexoelectricity in STO through energetic ion irradiation. As‐fabricated flexoelectric materials exhibit two advancements: the incidence‐dose‐dependent strength and the precise control over polarization orientation. By varying the mass and dose of incident ions, lattice strain can be well‐defined and enhanced up to ≈9.4%, following the quantized collision model employed in this study. Moreover, the lattice strain is characterized with the precise orientation from both macroscopic and microscopic perspectives. The well‐modulated flexoelectric configuration in ion irradiated STO is featured with out‐of‐plane polarization observed in PFM measurements. This research highlights the potential of ion irradiation for applications involving electric‐mechanical coupling effects and underscoring its promising role in future technological advancements.

## 4 Experimental Section

### Materials and Ion Irradiation

STO single crystals with the size of 10 × 10 × 0.5 mm^3^ are irradiated with 10 keV He^+^, C^+^, and P^+^ ions at doses up to 2 × 10^15^ ions cm^−2^ along the [100] orientation at room temperature under vacuum conditions of less than 4 × 10^−5^ Pa. The irradiation was performed by using a 200 kV ion implanter. In order to minimize the channeling effects, the sample surface was tilted by ≈7° relative to the incident ions during irradiation.

### Lattice Strain Derived from Symmetric X‐Ray Diffraction Measurements

The radiation‐induced lattice strains in all samples are evaluated from the diffraction profiles near the (200) lattice plane, which are measured using a high‐resolution thin film X‐ray diffractometer (PANalytical Empyrean) in *θ*‐2*θ* scan mode at room temperature. The wavelength of the X‐ray is 1.5406 Å (Cu Kα1 radiation). The quantitative strain‐depth profiles are derived from the simulation of the observed XRD patterns using the RaDMaX software.^[^ ^33^ ^]^ Furthermore, reciprocal spacing mappings are used to determine the presence or absence of radiation‐induced lattice strains in the in‐plane direction from the macroscopic view.

### Atomic‐Scale HRTEM Revealing Radiation‐Induced Lattice Strain

To reveal the lattice strains from the atomic scale, cross‐sectional TEM samples are prepared using a focused ion beam conducted on a ThermoFisher Helios 5UX from the STO sample irradiated with 10 keV C^+^ ions at a dose of 2 × 10^15^ ions cm^−2^. The accelerating voltage was lowered down to 1 kV to minimize additional damage, such as the undesired amorphous layer caused by Ga^+^ ions, which can seriously disturb the atomic‐scale observations. Meantime, the TEM lamella sample was prepared parallel to the (001) plane to conveniently observe the atom columns with the atomic scale with the [001] zone axis. Prior to the electron microscopy observation, the TEM sample is carefully cleaned by plasma. The electron microscopy was conducted on a spherical aberration corrected ThermoFisher Spectra 300 with an electron accelerating voltage of 300 kV.

### Piezoresponse Force Microscopy Analysis

To understand the electro‐mechanical coupling properties induced by ionic irradiation, piezoresponse force microscopy measurements are conducted on both unirradiated and C^+^ ion irradiated STO at doses of 1 × 10^14^ and 2 × 10^15^ ions cm^−2^. The measurements are performed using an Asylum Research Cypher ES scanning probe microscopy. A Pt/Ir‐coated conductive tip with an applied bias was used to image the electric polarization shape and to display the switch‐hysteresis loops. The vector mode was used to illustrate the switching of ferroelectric domains in both out‐of‐plane and in‐plane directions, simultaneously. In the PFM phase contrast switching test, two rectangles are electrically written with bias voltages of +8 and −8 V, respectively. Meantime, DARK SSPFM mode was conducted to obtain the piezoelectric hysteresis loop as a function of the voltage varying from +9 to −9V.

## Conflict of Interest

The authors declare no conflict of interest.

## Author Contributions

F.X.Z. and S.P.X. organize the research. S.N. performed ion irradiations, X‐ray diffraction, transmission electron microscopy, piezoelectric force microscopy, x‐ray photoelectron spectroscopy, electron backscattering diffraction, and Raman experiments. S.N., S.P.X., X.Y., Y.L.R., and Y.H.M. analyzed electric properties. P.F.Z. performed the ion irradiation work. S.P.X. developed the collision model. S.N., S.P.X., and F.X.Z. prepared the original manuscript. All authors discuss the results.

## Supporting information



Supporting Information

## Data Availability

The data that support the findings of this study are available from the corresponding author upon reasonable request.
