# Supplementary material for: Precise Tuning of Flexoelectricity in SrTiO3 by Ion Irradiation
Source: Adv Sci (Weinh). 2024 Dec 16;12(6):2411391. doi: 10.1002/advs.202411391 (PMC11809336; doi:10.1002/advs.202411391)
Supplement: Supplementary file 1 — Supporting Information [file ADVS-12-2411391-s001.docx]

**S1 Primary strain engineering strategies for centrosymmetric bulks**

Table S1 summarizes the diverse ways in literature to induce strain gradient in centrosymmetric materials ^[1]^, including interface engineering ^[2]^, external mechanical stress ^[3,4]^, nano engineering ^[5]^, element doping ^[6]^, and compositional gradient introduction ^[7]^. Besides element doping and epitaxial thin film which can induce strain gradient described in the main text, Gao *et al.* constructed strain gradients in the grain boundary. By imposing external stress on the single crystal of SrTiO_3_, Zubko *et al.* 3 demonstrated that the strain gradient in the bent crystals induces dielectric polarization.

**Table S1** Conventional methods for introducing flexoelectricity in centrosymmetric materials

| Materials | Methods | Strain gradient orientation ^α^ | Domain size | Ref. |
| --- | --- | --- | --- | --- |
| SrTiO_3_, LaAlO_3_ | interface engineering | 90° | several unit cells | ^[2]^ |
| SrTiO_3_, Si, TiO_2_ | external mechanical stress | undefined | undefined | ^[3,4]^ |
| LaAlO_3_ | Nano engineering | undefined | 3-4 unit cells | ^[5]^ |
| (Bi_1.5_Zn_0.5_)(Zn_0.5_Nb_1.5_)O_7_/Ag | element doping | undefined | undefined | ^[6]^ |
| SrTiO_3_-LaAlO_3_ | epitaxial thin film | undefined | undefined | ^[7]^ |
| Bulk SrTiO_3_ | ion irradiation | out-of-plane | tunable | This work |

^α^ The angle between strain gradient and its stimulating source.

**S2. Powder X-ray diffraction profiles of SrTiO_3_ single crystals**


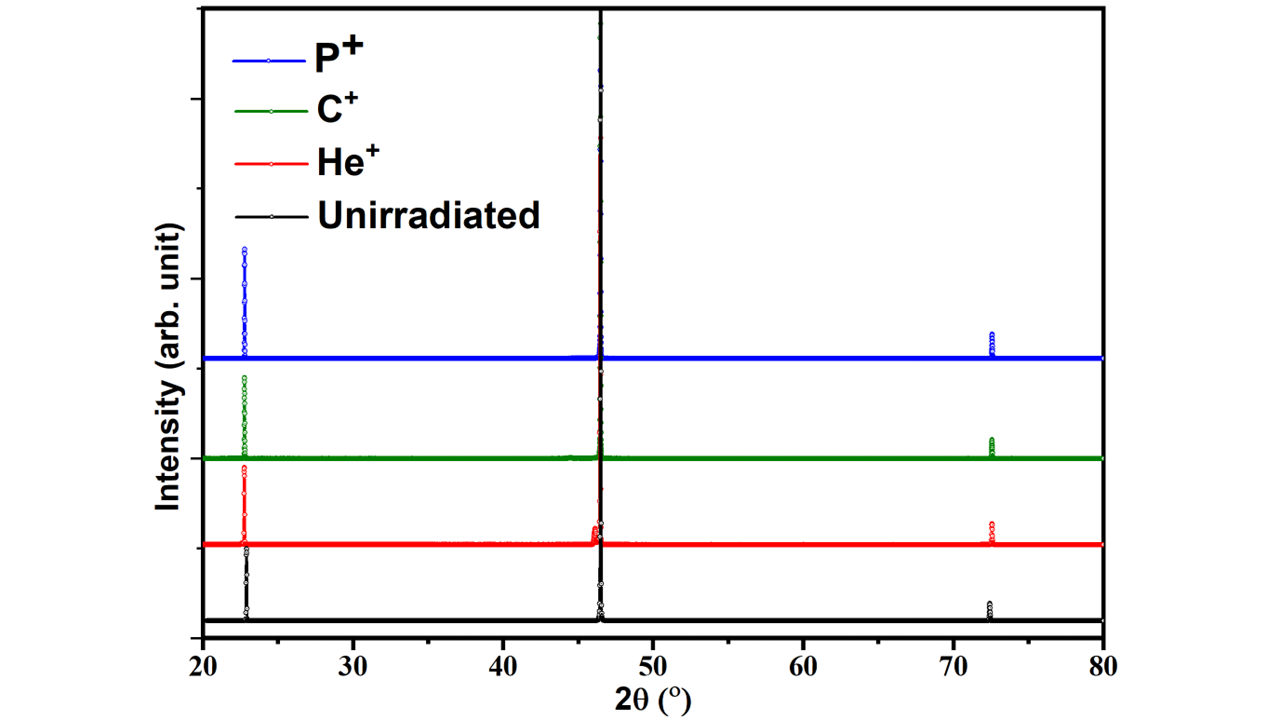


**Figure S1. XRD profiles of SrTiO_3_.** The intensity is shown in linear scale. The XRD results suggest that unirradiated STO and STO crystals irradiated with 10 keV ions of He^+^, C^+^ and P^+^ all have good single crystality and only strain-induced fringes observed in C+ and He+ irradiated samples.

**S3. PFM measurements on the unirradiated SrTiO_3_**

The electro-mechanical coupling in the unirradiated SrTiO_3_ is evidenced by piezoresponse force microscopy measurements. After pre-scanning from +8 to -8 V, the unirradiated SrTiO_3_ shows no reversal towards the out-of-plane direction in the phase-contrast image (Figure S2a). This is dramatically different from the ~80° phase inversion after electrically pre-polarization on the C^+^-ion-irradiated SrTiO_3_ (the dose of 2×10^15^ ions/cm^2^). Figure S2b shows the negligible phase reversal in the in-plane direction of unirradiated SrTiO_3_, which is consistent with the non-existence of piezoelectric effect in the in-plane direction on the C^+^-irradiated sample (see Fig. 5c). No electro-mechanical coupling is also confirmed by the non-existence of butterfly-shaped amplitude loops and polarization reversals in the out-of-plane direction (Figure S2c) and in-plane direction (Figure S2d).


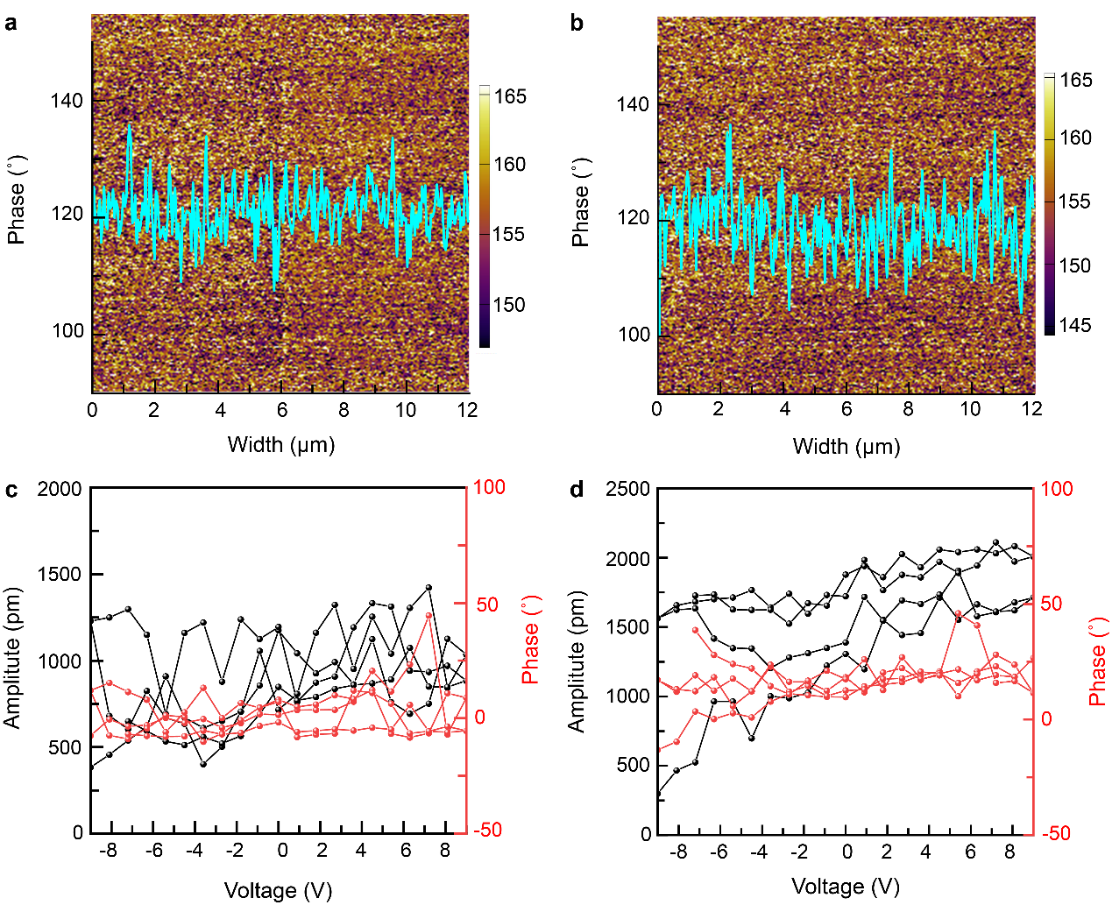


**Figure S2. PFM measurements of unirradiated SrTiO_3_ at room temperature.** Phase contrast images in (a) the out-of-plane and (b) the in-plane direction. The electric hysteresis loops analysis in (c) the out-of-plane and (d) the in-plane direction.

**S4. PFM measurements on the SrTiO_3_ with strain gradient of ~0.05%·nm^-1^**

Ion irradiated STO with strain gradient as low as ~0.05%·nm^-1^ can effectively induce flexoelectric properties. Figure S3a shows the PFM phase contrast pattern of C^+^ ions irradiated SrTiO_3_ with a low dose of 1×10^14^ ions/cm^2^ in the out-of-plane direction. This sample presents a polarization reversion of ~150°, of which a strain gradient is around 0.05%·nm^-1^. The yellow and dark regions illustrate the flexoelectric domains. However, in the in-plane direction, no stable polarization reversion is observed (Figure S3b).

Excited by using ±9 V voltage pulses, the C^+^ irradiation-induced strain gradient shows the polarization reversion of ~180° and the amplitude loop with a butterfly shape from the out-of-plane direction (Figure S3c). However, in the in-plane direction, no stable polarization reversion and butterfly-shaped amplitude loop are obtained in Figure S3d.


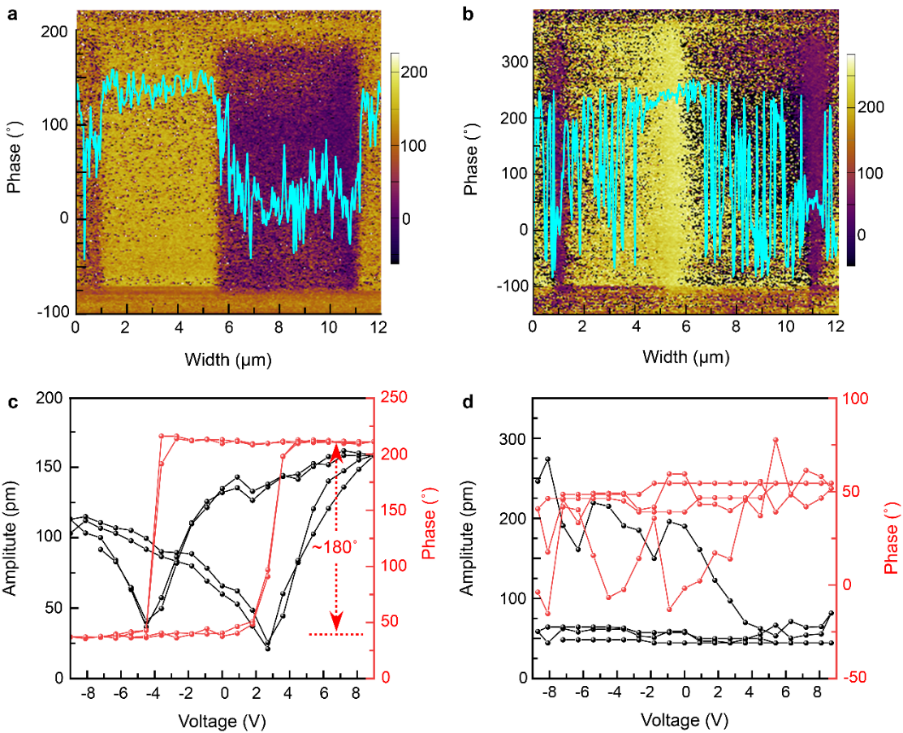


**Figure S3. PFM measurements of the irradiated SrTiO_3_. With strain gradient of 0.05%/nm** Phase contrast images in (a) the out-of-plane and (b) the in-plane direction. The electric hysteresis and amplitude spectroscopy loops in the (c) out-of-plane direction and (d) in-plane direction.

**S5. XPS analysis of the SrTiO3 irradiated by C+ ions with a dose of 2×10^15^ ions/cm^2^**

After irradiated with C^+^ ions, both Sr 3d and Ti 2p spectra in XPS measurements show additional peaks besides the main doublets in SrTiO_3_. Figures S4a and S4b show the full spectra of the unirradiated and C+ irradiated SrTiO_3_ at a dose of 2×10^15^ ions/cm^2^, respectively. Compared with the Sr 3d spectrum of the unirradiated sample (Figure S4c), the additional Sr 3d peaks shift to higher binding energy (Figure S4d), which possibly corresponds to the formation of SrO structure or metallic Sr structure ^[8]^. Different from the Ti 2p spectrum of the unirradiated sample (Figure S4e), the additional doublets in the C^+^-irradiated sample can be assigned to a lower oxidation state, *i.e.*, Ti^3+^, relative to the 4^+^ state ^[9]^ (Figure S4f). Thus, XPS measurements indicate that there is observable changes of the local structure for both Sr and Ti after irradiation.


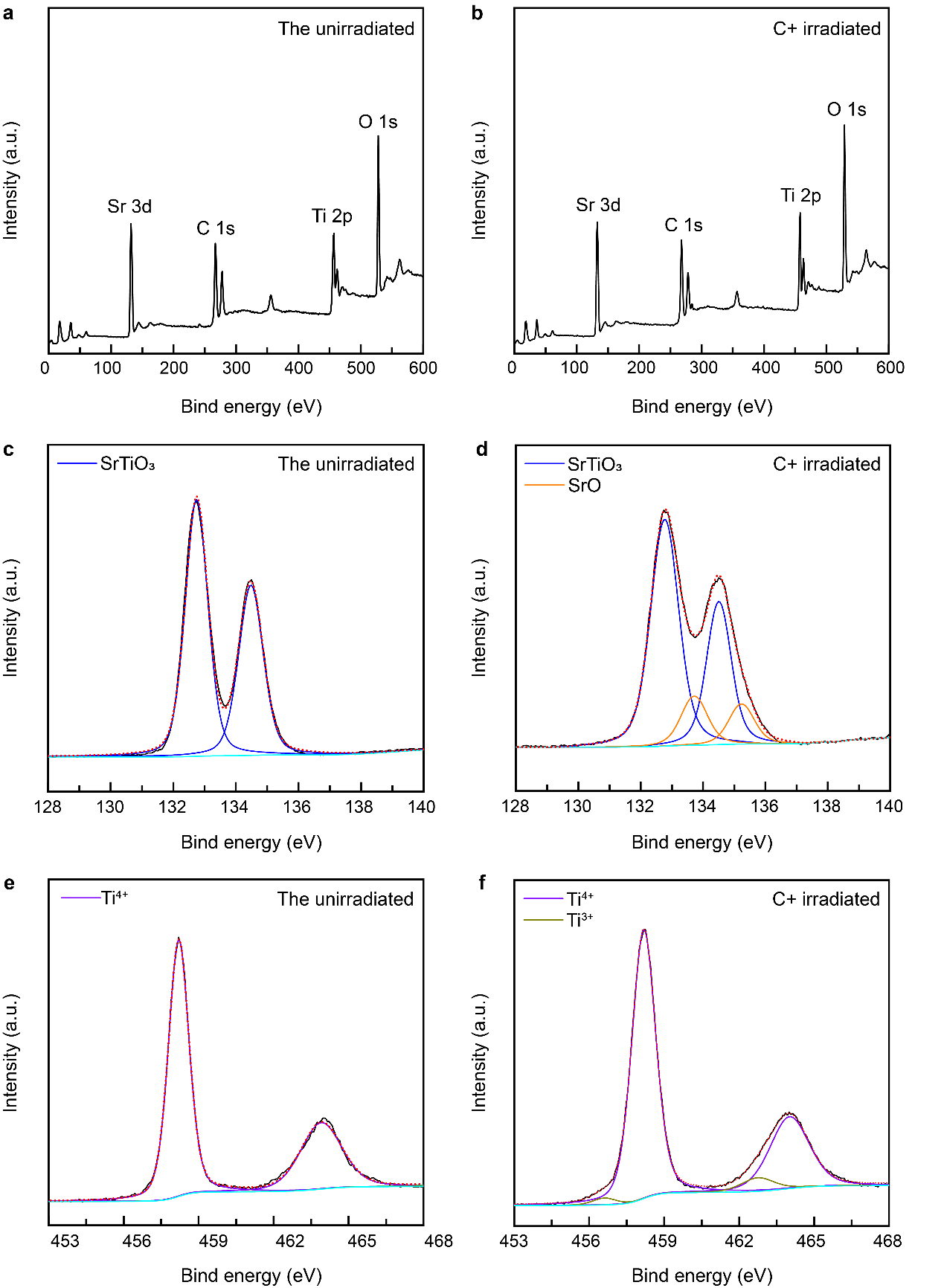


**Figure S4.** **XPS of the irradiated SrTiO_3_.** Full spectra of the (a) unirradiated and (b) irradiated samples. Sr 3d spectra of the (c) unirradiated and (d) irradiated samples. Ti 2p spectra of the (c) unirradiated and (d) irradiated samples.

**S6. Atomic-scale elemental mapping analysis of the C^+^ irradiated SrTiO_3_ with a dose of 2×10^15^ ions/cm^2^**

Although plenty of edge dislocations formed under C^+^ ions irradiation with a dose of 2×10^15^ ions/cm^2^, the lattice structures remain almost unchanged in the zone far away from dislocation. Figure S5a shows the atomic scale HAADF image from the C^+^ ion irradiated region where no edge dislocation exits. The Sr and Ti lattice structures almost completely match the intrinsic SrTiO_3_ structure, shown in the inserted figure at the top-right corner of Figure S5a, in which yellow and green balls correspond to Ti and Sr atoms, respectively. As the scattering intensity in the HAADF image is roughly proportional to the square of atomic number, *Z*, the brighter and bigger scattering sites in Figure S5a are Sr atoms, and the darker and smaller sites are Ti atoms.

Furthermore, the elemental mapping from the zone shown in Figure S5a also confirms the intact structure of Ti and Sr lattices (Figures S5b to S5d). Figure S5b illustrates the overlap mapping of Ti and Sr lattice structures. As is shown in Figure S5c and Figure S5d, the lattice spacings in both Ti and Sr sublattices in the [100] direction, *i.e.*, the ion incident direction, are 3.9 Å, consistent with the ideal SrTiO_3_ structure.


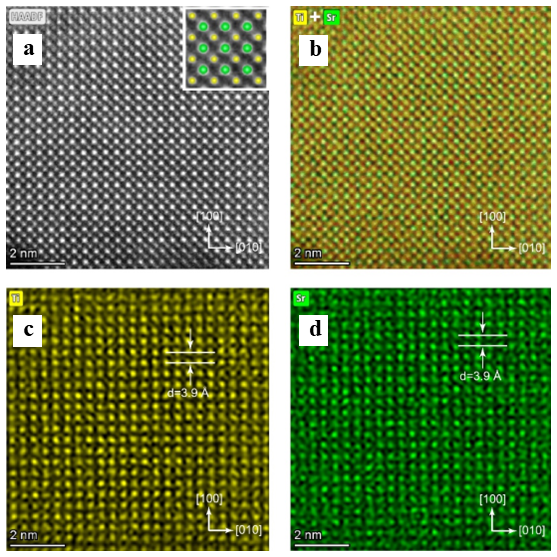


**Figure S5. Atomic scale elemental mapping from the position far away from a dislocation.** (a) HAADF. The elemental mapping of (b) overlap of Sr and Ti, (c) Ti and (d) Sr atoms.

**S7. EBSD and Raman analyses of C^+^ irradiated SrTiO_3_ at a dose of 2×10^15^ ions/cm^2^**

Although irradiated by 10 keV C+ ions with a dose of a dose of 2×10^15^ ions/cm^2^, the electron backscattered diffraction (EBSD) and Raman analyses demonstrate that the bulk SrTiO_3_ remains intact. Figure S6a and Figure S6b show the EBSD and the corresponding SEM image, respectively. The yellow dashed rectangle marks the irradiated zone, and others are unirradiated. The ESDB demonstrates that the substrate remains unchanged. The near compatibility of Raman spectra of the two samples shown in Figure S6c indicates that the bulk of the irradiated SrTiO_3_ was not broken, where the detecting depth of Raman scattering with 532 nm laser is ~2 µm ^[10]^.


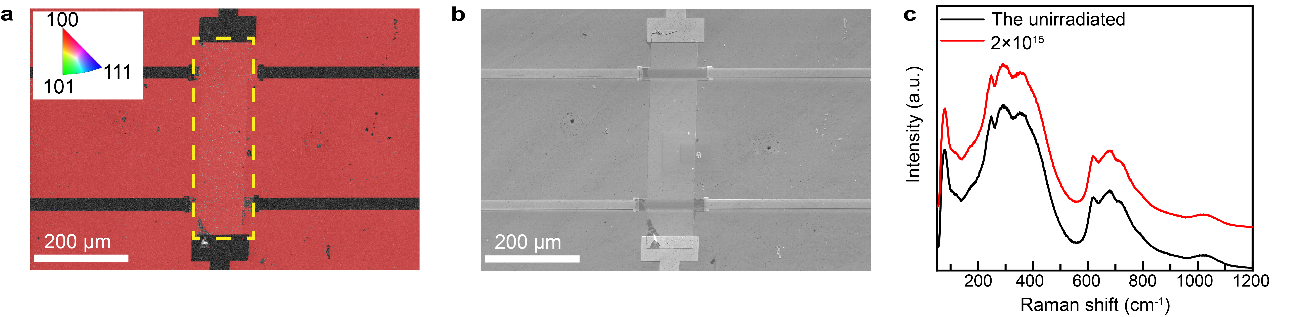


**Figure S6. Scanning electron microscopy and Raman analysis on the C^+^ irradiated SrTiO_3_.** (a) EBSD and (b) the corresponding SEM image on the irradiated zone (marked by the yellow dashed rectangle) and the resulting unirradiated zone. (c) Raman spectra of the irradiated and unirradiated SrTiO_3_.

**References**

[1] W. Hu, K. Kalantar-Zadeh, K. Gupta, C. P. Liu, *MRS Bull.* **2018**, *43*, 936.

[2] Z. L. Wang, *Adv. Mater.* **2012**, *24*, 4632.

[3] Y. Zhang, W. Jie, P. Chen, W. Liu, J. Hao, *Adv. Mater.* **2018**, *30*, 1707007.

[4] T. Li, S. Deng, H. Liu, S. Sun, H. Li, S. Hu, S. Liu, X. Xing, J. Chen, *Adv. Mater.* **2021**, *33*, 2008316.

[5] M. Wu, X. Zhang, X. Li, K. Qu, Y. Sun, B. Han, R. Zhu, X. Gao, J. Zhang, K. Liu, X. Bai, X. Z. Li, P. Gao, *Nat. Commun.* **2022**, *13*, 216.

[6] B. Wang, Y. Gu, S. Zhang, L. Q. Chen, *Prog. Mater. Sci.* **2019**, *106*, 100570.

[7] N. Li, R. Zhu, X. Cheng, H. J. Liu, Z. Zhang, Y. L. Huang, Y. H. Chu, L. Q. Chen, Y. Ikuhara, P. Gao, *Scr. Mater.* **2021**, *194*, 113624.

[8] X. Li, B. Han, R. Zhu, R. Shi, M. Wu, Y. Sun, Y. Li, B. Liu, L. Wang, J. Zhang, C. Tan, P. Gao, X. Bai, *Proc. Natl. Acad. Sci.* **2023**, *120*, e2213650120.

[9] K. J. Choi, M. Biegalski, Y. L. Li, A. Sharan, J. Schubert, R. Uecker, P. Reiche, Y. B. Chen, X. Q. Pan, V. Gopalan, L. Q. Che, D. C. Schlom, C. B. Eom, *Science (80-. ).* **2004**, *306*, 1005.

[10] J. Briscoe, S. Dunn, *Nanostructured Piezoelectric Energy Harvesters* (Eds.: J. Briscoe, S. Dunn), Publishing, Springer International **2014**.
